# Supplementary material for: Engineering an artificial catch bond using mechanical anisotropy
Source: Nat Commun. 2024 Apr 8;15:3019. doi: 10.1038/s41467-024-46858-9 (PMC11001878; doi:10.1038/s41467-024-46858-9)
Supplement: Supplementary file 1 — Supplementary Information [file 41467_2024_46858_MOESM1_ESM.pdf]

Supplementary Information

Engineering an artificial catch bond using mechanical anisotropy

Zhaowei Liu[#, 1, 2], Haipei Liu[1, 2], Andrés M. Vera[3], Byeongseon Yang[1, 2, 4, 5], Philip Tinnefeld[3], and Michael A. Nash[*, 1, 2, 4, 5, 6]

[1] Institute of Physical Chemistry, Department of Chemistry, University of Basel, 4058 Basel, Switzerland

[2] Department of Biosystems Science and Engineering, ETH Zurich, 4058 Basel, Switzerland

[3] Faculty of Chemistry and Center for NanoScience, Ludwig-Maximilians-Universität München, Munich, Germany

[4] Botnar Research Centre for Child Health, 4051 Basel, Switzerland

[5] National Center for Competence in Research (NCCR) Molecular Systems Engineering, 4058 Basel, Switzerland

[6] Swiss Nanoscience Institute, 4056 Basel, Switzerland

[#] Current address: Department of Bionanoscience, Delft University of Technology, Delft, the Netherlands

[*] Correspondence to: michael.nash@bsse.ethz.ch

Supplementary information

Supplementary figures

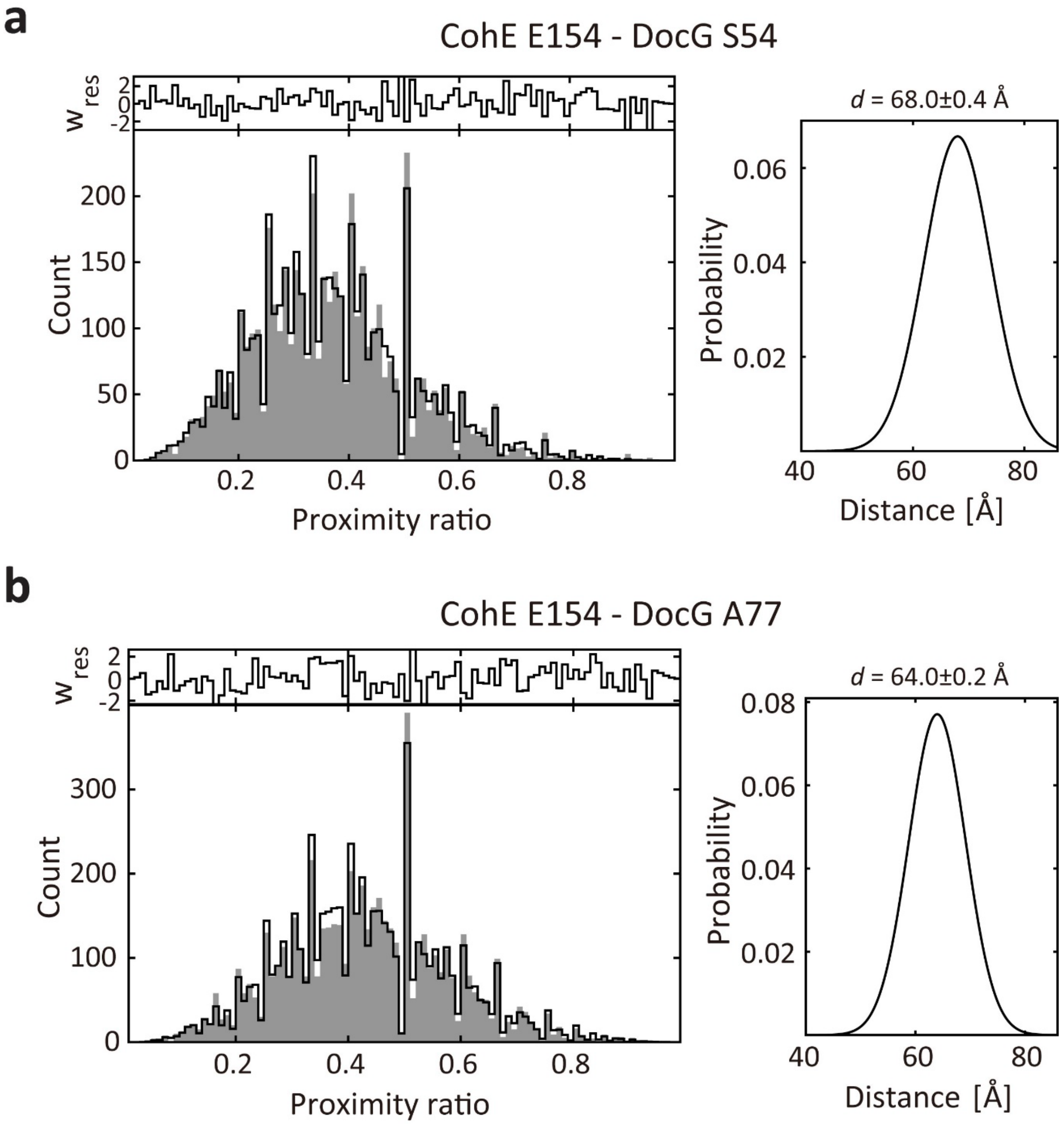

Figure S1 Photon density analysis (PDA) results. Left panel: simulated proximity ratio histograms of CohE E154 - DocG S54 measurement **(a)** and CohE E154 - DocG A77 measurement **(b)**. Right panel: probability density distribution of simulated donor-acceptor distance between CohE E154 and DocG S54 **(a)** and CohE E154 and DocG A77 **(b)**.

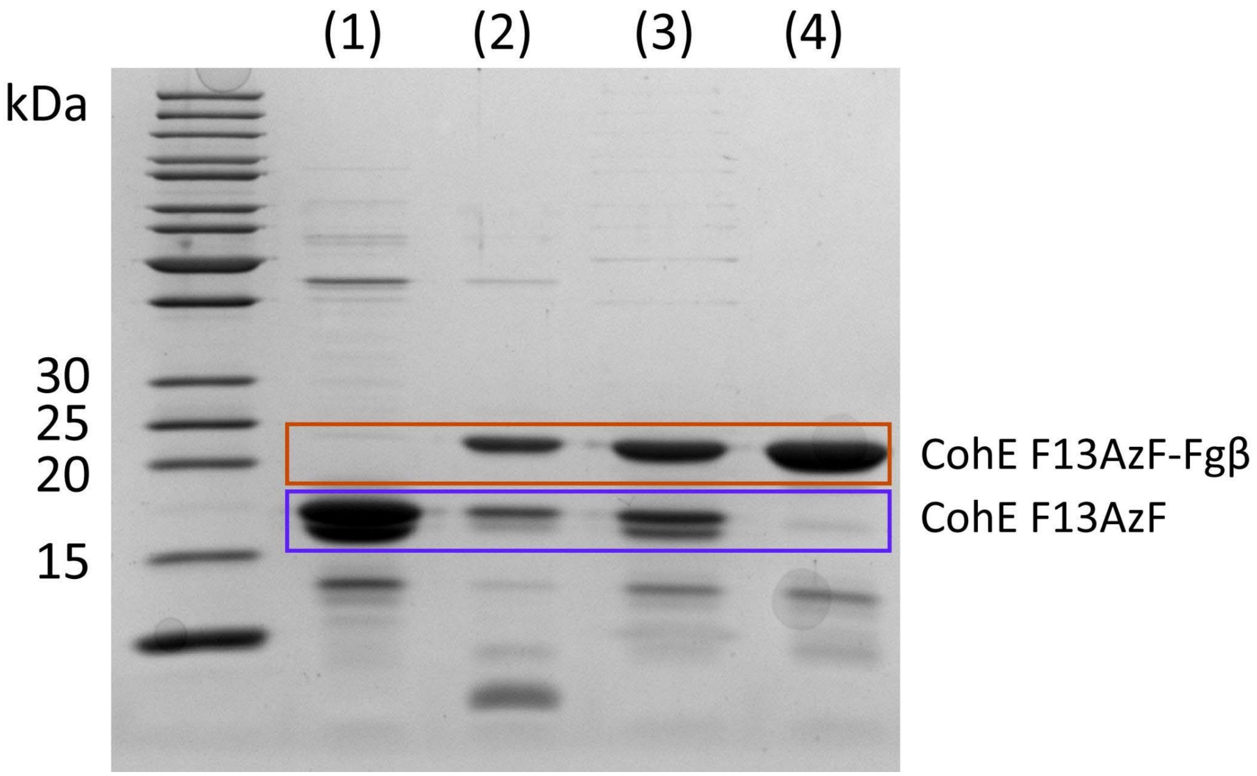

Figure S2 SDS-PAGE showing conjugation of Fgβ to Coh F13AzF mutant. The Coh F13AzF mutant (Lane 1) was conjugated with Fgβ peptide. The reaction product (Lane 2) was subsequently purified using a size-exclusion column to remove the excess Fgβ peptide (Lane 3), and a Strep-trap column to remove unconjugated Coh F13AzF (Lane 4).

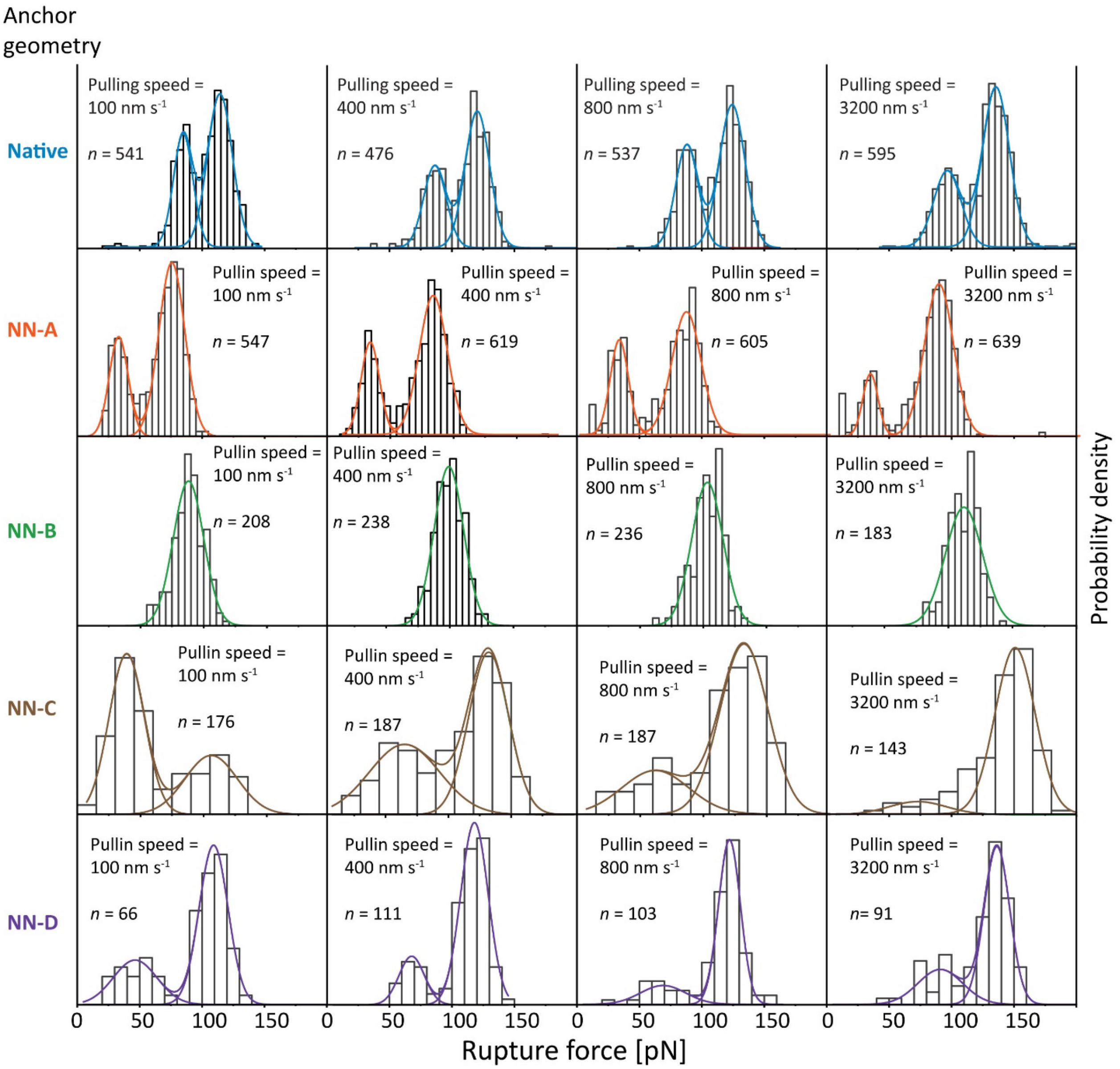

Figure S3 Rupture force histograms measured at different anchor geometries and different pulling speeds. The histograms were fitted with one (anchor geometry NN-B, pulling from CohE N-terminus and DocG N-terminus) or two (other anchor geometries) Gaussian peaks.

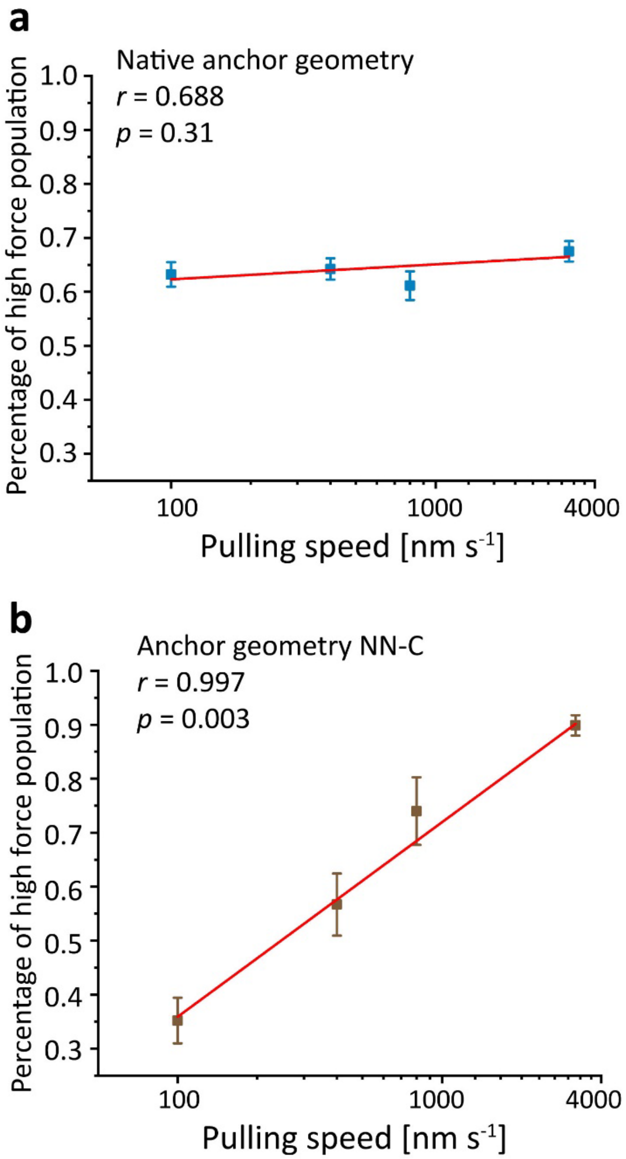

Figure S4 Linear regression between the percentage of high force population and the logarithm of pulling speed. Only anchor geometries exhibiting bimodal rupture force distribution are shown: native anchor geometry **(a)**, and non-native anchor geometries NN-A **(b)**, NN-C **(c)**, and NN-D **(d)**. For each anchor geometry, the prevalence of the high force population measured at different pulling speeds are plotted against the logarithm of the pulling speed and fitted linearly. The Pearson's correlation coefficients r and the p-values of Analysis of Variance (ANOVA) test are shown for each anchor geometry and summarized in Table S2. Only anchor geometry NN-C **(panel c)** has a p-value<0.05, meaning that the slope of linear fitting is significantly larger than zero.

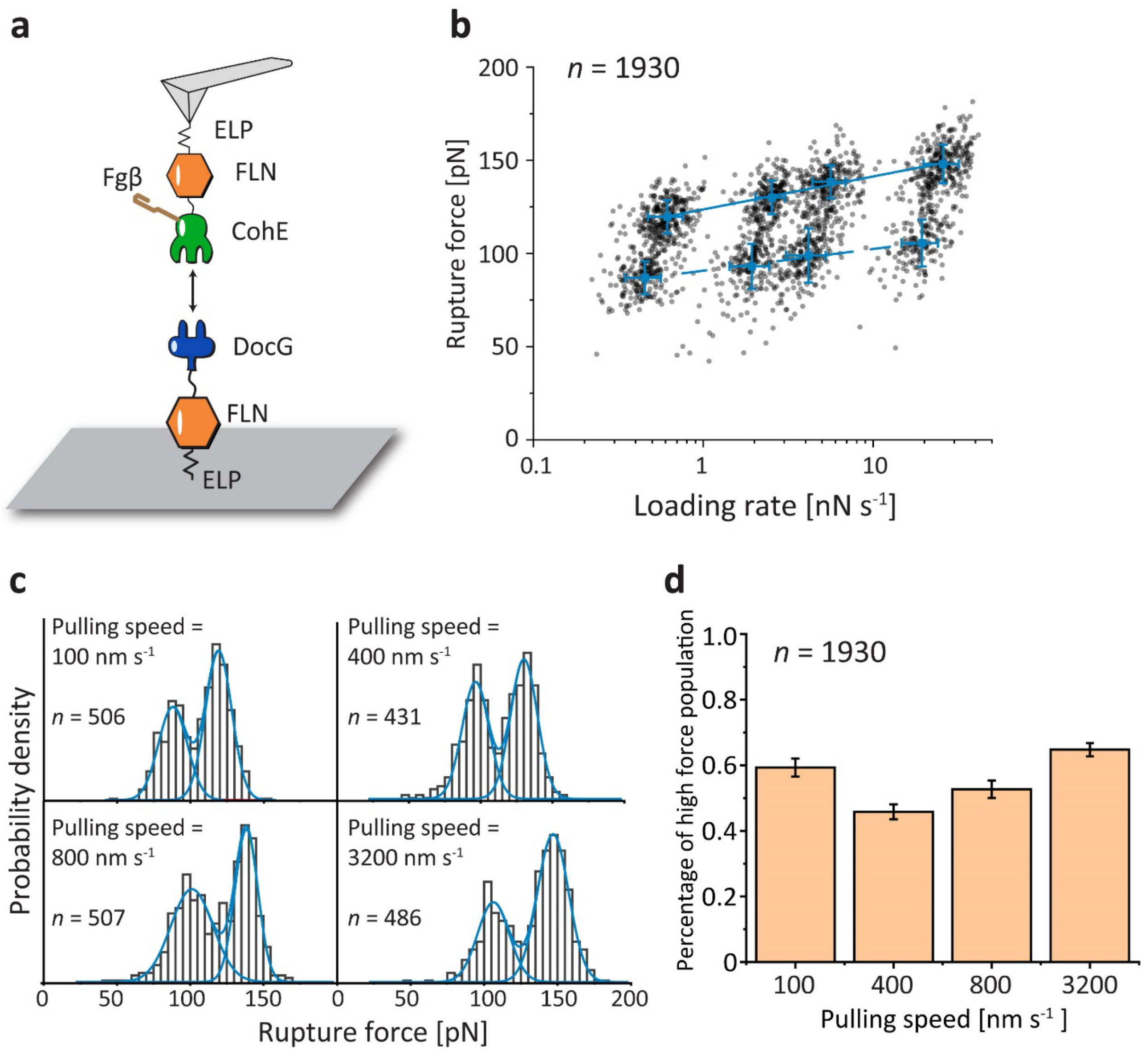

Figure S5 Native anchor geometry measurement of DocG:CohE (F13-Fgβ). a: AFM-SMFS experimental setup. The residue F13 of CohE in CohE-FLN-ELP-ybbr construct was replaced by azido-phenylalanine and conjugated with Fgβ peptide, and the construct was immobilized on the AFM tip. ybbr-ELP-FLN-DocG construct was immobilized on the glass surface. The rupture force of DocE:CohG (F13-Fgβ) complex was measured at the native anchor geometry and at different pulling speeds. **b:** The force-loading rate plot of DocG:CohE (F13-Fgβ). The average rupture forces of the high force and low force pathways measured at four different pulling speeds were linearly fitted against loading rate to extract energy landscape parameters. The *n* number represents the total number of single protein complexes measured. **c:** Rupture force histograms of DocG:CohE (F13-Fgβ) at different pulling speeds. The *n* numbers represent the number of single protein complexes measured at each pulling speed. **d:** Fraction of high force pathway at different pulling speeds. The *n* number represents the total number of single protein complexes measured.

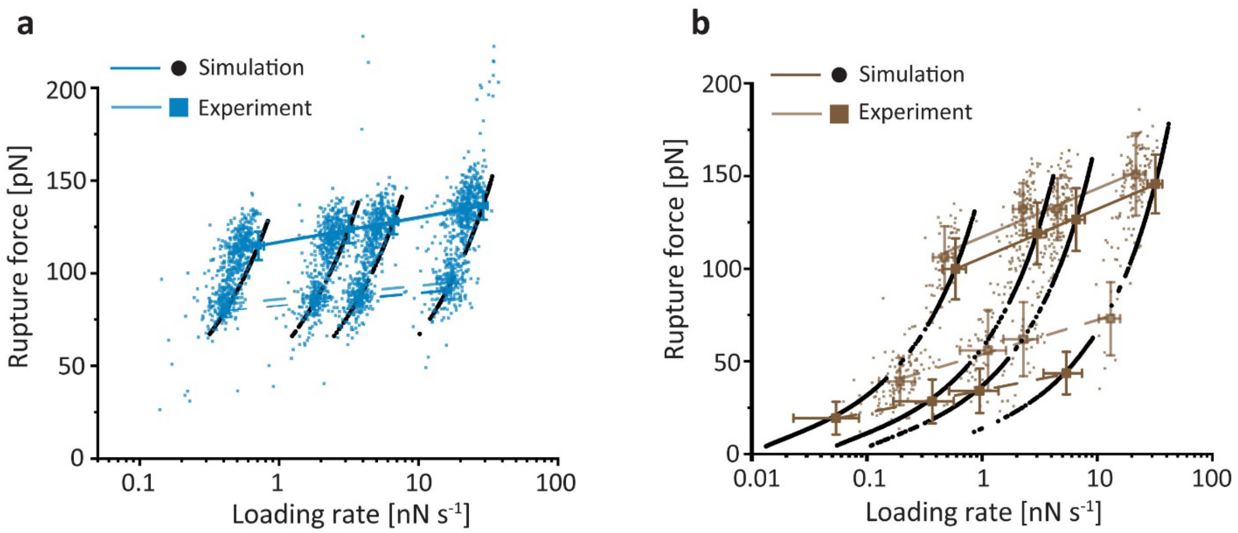

Figure S6 Overlay of force-loading rate plots of experimental and simulation results. The Monte Carlo simulation results for the force-loading rate relationship at the native **(a)** and catch **(b)** anchor geometries are overlayed with the corresponding experimental results. The simulation results are shown in black dots and the experimental data are shown in blue (native anchor geometry) and brown (catch anchor geometry) squares. The Bell-Evans fitting results are shown in solid line (high-force population) and dashed line (low-force population).

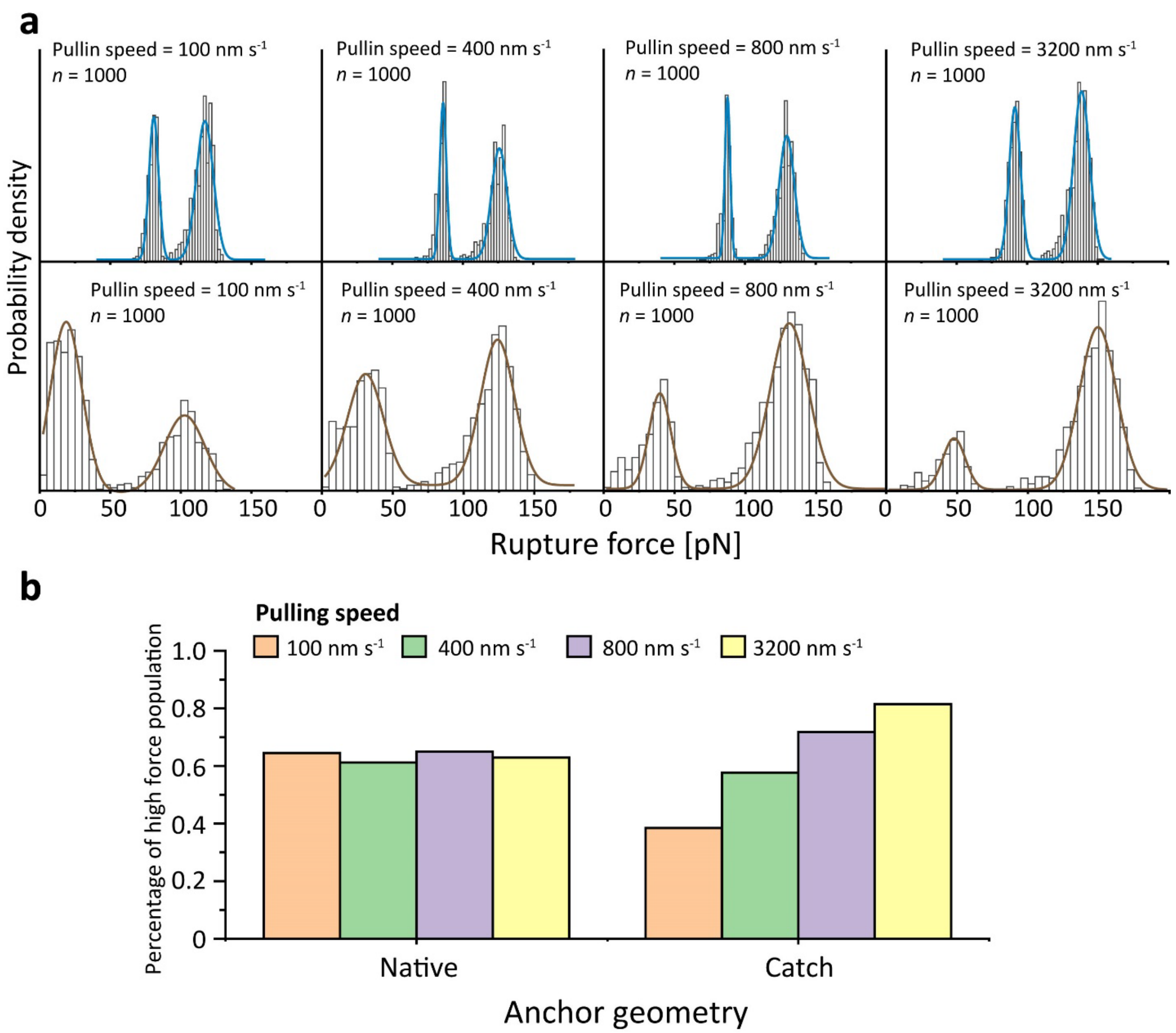

Figure S7 Monte Carlo simulation rupture force histograms. a: Rupture force histograms of DocG:CohE at native anchor geometry (upper panels) and catch anchor geometry (corresponding to the non-native anchor geometry NN-C, lower panels). Each histogram was fitted in two Gaussian peaks. **b:** The prevalence of high rupture force population at different pulling speeds in Monte Carlo simulation. At the catch anchor geometry, the prevalence of high force population increases with increasing pulling speed (ANOVA test of linear regression $p < 0.05$, see **Table S2**), which is in contrast to the native anchor geometry.

Supplementary tables

Table S1 Equilibrium affinity of the Doc:Coh complex

	Wild-type CohE	CohE F13AzF-Fgβ	CohE K87AzF-Fgβ
Dissociation constant [nM]	14±4	40±8	19±10

Table S2 Pearson's correlation coefficients between logarithm of pulling speed and prevalence of high force unbinding pathway

**Anchor geometry**	**Pearson's correlation coefficient *r***	***p*-value of ANOVA test**
Native (experiment)	0.688	0.31
Native (Monte Carlo simulation)	-0.196	0.804
NN-A (experiment)	0.773	0.227
NN-B (experiment)	N/A	N/A
NN-C (experiment)	0.997	0.003
NN-C (Monte Carlo simulation)	0.982	0.02
NN-D (experiment)	0.420	0.580

Table S3 Unbinding energy landscape parameters calculated from Monte Carlo simulations

Anchor geometry	Pathway	log(k_0)	$\Delta x^{\ddagger}$ [nm]
Native (slip)	High force	-6.5±0.1	0.720±0.009
	Low force	-9.9±0.2	1.42±0.02
NN-C (catch)	High force	-1.80±0.09	0.368±0.008
	Low force	-0.6±0.1	0.80±0.03

Supplementary note

Amino acid sequences

Color code:

ybbr tag, His tag, ELP linker, FLN, DocG, CohE, AzF

ybbr-His-ELP-FLN-DocG

MGTDSLEFIASKLAHHHHHHWGSGHGVGVPGMGVPGVGVPGVGVPGVGVPGVGVPGVGVPG
VGVPGVGVPGEGVPGEGVPGVGVPGMGVPGVGVPGVGVPGVGVPGVGVPGVGVPGVGVPGV
GVPGEGVPGEGVPGVGVPGMGVPGVGVPGVGVPGVGVPGVGVPGVGVPGVGVPGVGVPGEG
VPGEGVPGWPSGSADPEKSYAEGPGLDGGESFQPSKFKIHAVDPDGVHRTDGGDGFVVTIEGPA
PVDPVMVDNGDGTYDVEFEPKEAGDYVINLTLDGDNVNGFPKTVTVKPAPGSGSGSGSGVGDS
LLRGDVDLDGDVDVADAVAVLQASAEQMVTGENPLSKDARFGADVNDDSRVDVSDAVLILQY
SSMKIANPDADWDDLLG

CohE-FLN-ELP-His-ybbr

ADGAAKLSMDQKFAEPGETVEIALNLENFDASWTGLEFLVNYDPKLEVALDGAGDIDYSYGDAI
GAMGKKISVGGAISKDLTADGLKGFAFAWGTATAISGNGQLGVFKFTVPADAQPGDEFPVNLTV
NVGSFIDANKENIPFETVNGWIKIKEEGSGSGSGSADPEKSYAEGPGLDGGECFQPSKFKIHAVDP

DGVHRTDGGDGFVVTIEGPAPVDPVMVDNGDGTYDVEFEPKEAGDYVINLTLDGDNVNGFPKT
VTVKPAPGSGSGSHGVGVPGMGVPGVGVPGVGVPGVGVPGVGVPGVGVPGVGVPGVGVPGEG
VPGEGVPGVGVPGMGVPGVGVPGVGVPGVGVPGVGVPGVGVPGVGVPGVGVPGEGVPGEGVP
GVGVPGMGVPGVGVPGVGVPGVGVPGVGVPGVGVPGVGVPGVGVPGEGVPGEGVPGWRGHH
HHHHGSDSLEFIASKLA

DocG-FLN-ELP-His-ybbr

GVGDSLLRGDVDLDGDVDVADAVAVLQASAEQMVTGENPLSKDARFGADVNDDSRVDVSDA
VLILQYSSMKIANPDADWDDLLGGSGSGSADPEKSYAEGPGLDGGECFQPSKFKIHAVDPDGVH
RTDGGDGFVVTIEGPAPVDPVMVDNGDGTYDVEFEPKEAGDYVINLTLDGDNVNGFPKTVTVK
PAPGSGSGSHGVGVPGMGVPGVGVPGVGVPGVGVPGVGVPGVGVPGVGVPGVGVPGEGVPGE
GVPGVGVPGMGVPGVGVPGVGVPGVGVPGVGVPGVGVPGVGVPGVGVPGEGVPGEGVPGVG
VPGMGVPGVGVPGVGVPGVGVPGVGVPGVGVPGVGVPGVGVPGEGVPGEGVPGWRGHHHHH
HGSDSLEFIASKLA

ybbr-His-ELP-FLN-CohE

MGTDSLEFIASKLAHHHHHHWGSGHGVGVPGMGVPGVGVPGVGVPGVGVPGVGVPGVGVPG
VGVPGVGVPGEGVPGEGVPGVGVPGMGVPGVGVPGVGVPGVGVPGVGVPGVGVPGVGVPGV
GVPGEGVPGEGVPGVGVPGMGVPGVGVPGVGVPGVGVPGVGVPGVGVPGVGVPGVGVPGEG
VPGEGVPGWPSGSADPEKSYAEGPGLDGGESFQPSKFKIHAVDPDGVHRTDGGDGFVVTIEGPA
PVDPVMVDNGDGTYDVEFEPKEAGDYVINLTLDGDNVNGFPKTVTVKPAPGSGSADGAAKLSM
DQKFAEPGETVEIALNLENFDASWTGLEFLVNYDPKLEVALDGAGDIDYSYGDAIGAMGKKISV
GGAISKDLTADGLKGFAFAWGTATAISGNGQLGVFKFTVPADAQPGDEFPVNLTVNVGSFIDAN
KENIPFETVNGWIKIKEE

CohE (F13AzF)-FLN-ELP-His-ybbr

ADGAAKLSMDQKAzFAEPGETVEIALNLENFDASWTGLEFLVNYDPKLEVALDGAGDIDYSYG
DAIGAMGKKISVGGAISKDLTADGLKGFAFAWGTATAISGNGQLGVFKFTVPADAQPGDEFPVN
LTVNVGSFIDANKENIPFETVNGWIKIKEEGSGSGSGSADPEKSYAEGPGLDGGECFQPSKFKIHA
VDPDGVHRTDGGDGFVVTIEGPAPVDPVMVDNGDGTYDVEFEPKEAGDYVINLTLDGDNVNGF

PKTVTVKPAPGSGSGSHGVGVPGMGVPGVGVPGVGVPGVGVPGVGVPGVGVPGVGVPGVGVP
GEGVPGEGVPGVGVPGMGVPGVGVPGVGVPGVGVPGVGVPGVGVPGVGVPGVGVPGEGVPGE
GVPGVGVPGMGVPGVGVPGVGVPGVGVPGVGVPGVGVPGVGVPGVGVPGEGVPGEGVPGWR
GHHHHHHGSDSLEFIASKLA

CohE (F13AzF)-FLN-ELP-His-ybbr

ADGAAKLSMDQKAzFAEPGETVEIALNLENFDASWTGLEFLVNYDPKLEVALDGAGDIDYSYG
DAIGAMGKKISVGGAISKDLTADGLKGFAFAWGTATAISGNGQLGVFKFTVPADAQPGDEFPVN
LTVNVGSFIDANKENIPFETVNGWIKIKEEGSGSGSHHHHHH

CohE (K87AzF)-FLN-ELP-His-ybbr

ADGAAKLSMDQKFAEPGETVEIALNLENFDASWTGLEFLVNYDPKLEVALDGAGDIDYSYGDAI
GAMGKKISVGGAISKDLTADGLAzFGFAFAWGTATAISGNGQLGVFKFTVPADAQPGDEFPVNL
TVNVGSFIDANKENIPFETVNGWIKIKEEGSGSGSHHHHHH
